# The Importance of Bridge Jobs in America Today

**DOI:** 10.1093/geroni/igaa057.2938

**Published:** 2020-12-16

**Authors:** Kevin Cahill

**Affiliations:** Boston College, Boston, Iowa, United States

## Abstract

Gradual retirement, consisting of phased retirement, bridge employment, and reentry, has been a persistent feature of older Americans’ work decisions for decades. This persistence is remarkable in light of the many changes to the retirement landscape that have taken place since the mid-1980s, including changes to Social Security incentives, private-sector pensions, and macroeconomic volatility. Our paper addresses two topics related to these retirement transitions. First, using 26 years of data from the Health and Retirement Study (HRS), we quantify the prevalence of bridge employment among the initial group of HRS respondents. For the first time, bridge job prevalence can be calculated for a large cohort of older Americans who have completed their retirement transitions. Second, we comment on what these findings mean for policymakers, with respect to income inequality later in life, barriers to retirement pathways, and public-private sector differences, and what role public employers might be able to play.

